# A New Specific for Rheumatism

**Published:** 1873-06

**Authors:** 


### ARTICLE XIII.

### *A New Specific for Rheumatism.*

Rheumatism, notwithstanding that it is one of the most obstinate diseases, some forms of which baffle the skill of the most eminent physicians, is, from a medical point of view, highly interesting; the late Dr. Valentine Mott used even to say that "it is one of the beauties of rheumatism that it shows itself in such a great variety of forms." It is a fact well known among the medical profession that many rheumatic patients, in the impatience produced by their affliction, change from one physician to another; at length the disease has run its course, the patient gets well, and the last doctor whom they then happen to have, earns the credit of the cure.

Without intending to trespass on the domain of the physician, it may be well to give, for the benefit of all, some information concerning the nature and treatment of this malady.

As it is a constitutional disease, proper diet and close attention to the general health are of more benefit than local applications, which may be useful in exceptional cases, but generally they give only temporary relief, and often drive the pain from one part of the body to another. In all cases of this disease, the blood is in an abnormal condition, and may be considered to be poisoned; persons who live high (which means, live on rich and highly nitrogenized food) are apt to have this disease in a peculiar form, which is commonly called gout, of which the chief seat is in the joints. A lower mode of diet is then advisable. Persons who live low and get this disease by exposure, combined with over fatigue, are apt to suffer from the so called chronic form chiefly seated in the muscles, and in these cases, the system may suffer from one or two opposite causes, an excess of either alkali or acid, which, when neutralized, ends the disease. Hence the curious and formely unexplained fact that sometimes acid treatment, as with lemon juice, and at other times alkaline treatment, as with Rochelle Salts, etc., has produced a cure.

There is one very severe form of rheumatism called acute or inflammatory, which is a most formidable disease, and which in olden times was treated by blood letting. This disease has the remarkable feature of suddenly leaving one part of the body to appear in another. If, by blood letting, the heart receives a sudden shock by the withdrawal of a quantity of blood, the malady is very apt to settle there and produce disease of the heart, which is a very common cause of death among persons who once have been treated for rheumatism by blood letting. The latter operation relieves the patient; but, considering the often fatal results, it is now abandoned by all enlightened physicians, and the treatment by colchicum wine and opiates is used instead. Besides the derivatives of opium, morphine and codeine, (see page 273 of our current volume), sal ammoniac has been often praised as an effective remedy when others failed; but perhaps these derive their efficiency from their similarity to a new substance, a derivative of opium and ammonia, which has recently been found as effective a specific against rheumatism as quinine is against fever and ague. This substance is propylamin. It is a volatile, watery liquid, with a strong odor of herring pickle, and was found by Dr. Winckler in distilling a watery extract of ergot with potassa, also in distilling cod liver oil with ammonia. But the most effective way of obtaining this substance is that of Werthein, who prepared it by the decomposition of narcotine and codeine by alkalies. Its name is based on its chemical composition; it is a combination of the third member of the hydrocarbon series (methyl, ethyl, propyl, amyl, etc.) with a derivative of the ammonia (amidogen, mentioned on pages

20 and 144 of our current volume.) There is, however, still some doubt about its true chemical composition, so that some chemists suppose it to be trimethylamin; in the mean time, its specific effect on most forms of rheumatism has been established. By taking five drops in a tablespoonful of peppermint water every two hours, the pains usually abate after twelve doses.—*Scientific American.*

